# The Late Professor Mendeléeff

**Published:** 1907-02-16

**Authors:** 


### THE LATE PROFESSOR MENDELÉEFF.

One of the most celebrated exponents of modern physical science has passed away by the death of Professor Mendeléeff. To medical men his work does not directly appeal, except as an instance of the value of *a priori* reasoning as a guide to the discovery of fact. The name of the great Russian chemist will always be known as that of the enunciator of the so-called periodic law. One of the first fruits of the atomic theory which we owe to Darton was the determination of the atomic weights of all the then known elements. These weights once determined, physicists began to apply themselves to arranging the elements in series, their places being determined by fixed mathematical relationships found to exist between their respective atomic weights. Mendeléeff succeeded in doing this; but in the serial order he suggested for the elements there were several gaps. So convinced was he of the accuracy of his reasoning that he assigned to it the validity of a law, and predicted that future research would demonstrate the existence of elements possessing the atomic weights, and associated chemical properties required by the then hypothetical entities to which he had assigned the vacant places in his series. Precisely what he predicted happened; subsequently elements were actually discovered possessing the above properties, and thus now the gaps in his serial arrangement of the elements are no longer filled by hypothetical but by actual substances.

